# Tuning Earth System Models Without Integrating to Statistical Equilibrium

**DOI:** 10.1029/2024MS004230

**Published:** 2024-11-26

**Authors:** Timothy DelSole, Michael K. Tippett

**Affiliations:** ^1^ Department of Atmospheric, Oceanic, and Earth Sciences George Mason University Fairfax VA USA; ^2^ Department of Applied Physics and Applied Mathematics Columbia University New York NY USA

**Keywords:** model tuning, kalman filter, autoregressive models

## Abstract

This paper proposes algorithms for estimating parameters in Earth System Models (ESMs), specifically focusing on simulations that have not yet achieved statistical equilibrium and display climate drift. The basic idea is to treat ESM time series as outputs of an autoregressive process, with parameters that depend on those of the ESM. The maximum likelihood estimate of the parameters and the associated uncertainties are derived. This method requires solving a nonlinear system of equations and often results in unsatisfactory parameter estimates, especially in short simulations. This paper explores a strategy for overcoming this limitation by dividing the estimation process into two linear phases. This algorithm is applied to estimate parameters in the convection scheme of the Community Earth System Model version 2 (CESM2). The modified algorithm can produce accurate estimates from perturbation runs as short as 2 years, including those exhibiting climate drift. Despite accounting for climate drift, the accuracy of these estimates is comparable to that of algorithms that do not. While these initial results are not optimal, the autoregressive approach presented here remains a promising strategy for model tuning since it explicitly accounts for climate drift in a rigorous statistical framework. The current performance issues are believed to be technical in nature and potentially solvable through further investigation.

## Key Points

- Algorithms are proposed for estimating parameters in Earth System Models using simulations that have not reached statistical equilibrium
- The algorithms are based on autoregressive models with parameters that depend on those of the Earth System Model
- One of the methods generates accurate parameter estimates from perturbation runs that are as short as 2 years and exhibit climate drift

## 1 Introduction

Earth‐System Models (ESMs) are computationally expensive to run and therefore challenging to tune. Perhaps the most comprehensive approach to tuning is to incorporate parameter estimation into data assimilation (e.g., see the reviews by Ruis et al. (2013) and Zhang et al. (2020)). However, this approach is rarely used for ESM tuning due to its high computational cost. Instead, most algorithms for automated tuning algorithms treat the problem as a regularized inverse problem, where parameter values are optimized by minimizing a cost function that quantifies the discrepancies between model simulations and observational data. Most cost functions measure the difference in first‐order moments (Annan et al., 2005; Huang et al., 2022; Huerta et al., 2008). For example, the cost function may measure the difference in mean temperature or mean precipitation. We call these algorithms *moment‐based*, to distinguish them from data‐assimilation type algorithms that are based on instantaneous states.

A significant limitation of moment‐based algorithms arises from the temporal dynamics of ESMs. After parameters are adjusted, the ESM evolves toward a new equilibrium over a time scale of decades to centuries, a phenomenon termed climate drift. However, due to computational constraints, tuning usually involves shorter simulations, with substantial parts of those runs consisting of a transient drift toward an unknown equilibrium. The statistics of a drifting simulation differ from those at statistical equilibrium. Therefore, if the statistics of a drifting simulation are tuned to match observations, the resulting ESM will not match the observed statistics when it is integrated to statistical equilibrium, leading to biases in the ESM statistics.

In addition, uncertainty estimates from moment‐based algorithms are valid only if simulated and observational moments are computed from time series of the same length. When this is not the case, the resulting moments have different levels of sampling variability, complicating uncertainty assessments. Requiring time series to be of the same length often translates into disregarding some portion of the observations. For example, while observational data sets typically span 50–100 years, computational limitations usually restrict perturbation runs to a few years up to a decade or two. Consequently, adhering to equal length time series means utilizing only about a decade's worth of observational data. With fixed computational resources, adding more tunable parameters requires increasing the number of runs to adequately explore parameter space, which in turn necessitates shorter runs, leading to the use of even less observational data and further exacerbating biases from climate drift.

A natural idea for dealing with climate drift is to discard the initial years exhibiting the most pronounced drift, and use the remaining years for parameter tuning. However, discarding data wastes resources and overlooks potentially useful information. Our goal is to develop and test methodologies that account for drift and enable use of the full data set, regardless of whether runs have reached statistical equilibrium. Leveraging information from climate drift might significantly shorten the length of simulations required for effective tuning, speeding up the tuning process.

In this paper, we propose tuning algorithms that require neither statistical equilibrium nor time series of the same length. Our strategy is to assume output from climate models are realizations from an autoregressive (AR) process. The fact that this strategy can produce accurate parameter estimates even as the ESM drifts to a new equilibrium can be illustrated with a simple example. Consider the AR(1) model

(1) $$y_{t}=\phi y_{t-1}+k+w_{t},$$

where ϕ is a scalar in the interval (−1,1), yielding a stable model, k is a constant, and $w_{t}$ is white noise with zero mean and variance $\sigma_{w}^{2}$. For asymptotically long times, it is easily shown (DelSole & Tippett, 2022) that solutions approach a stationary distribution with the climatological mean and climatological variance

(2) $$E\left[y_{t}\right]=\frac{k}{1-\phi }\;\text{and}\;var\left[y_{t}\right]=\frac{\sigma_{w}^{2}}{1-\phi^{2}}.$$

**Figure 1:**
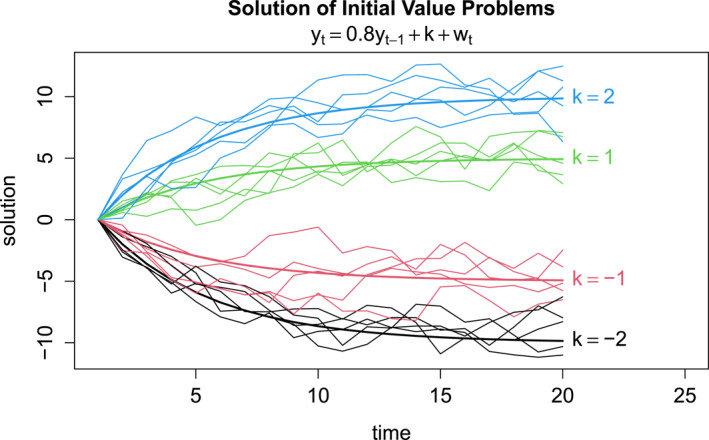
Solutions of the AR(1) model $y_{t}=0.8y_{t-1}+k+w_{t}$ for different values of k and different realizations of $w_{t}$, each initialized at the same value $y_{1}=0$. The thick curve shows the population mean and the thin curves show individual realizations.

**Figure 2:**
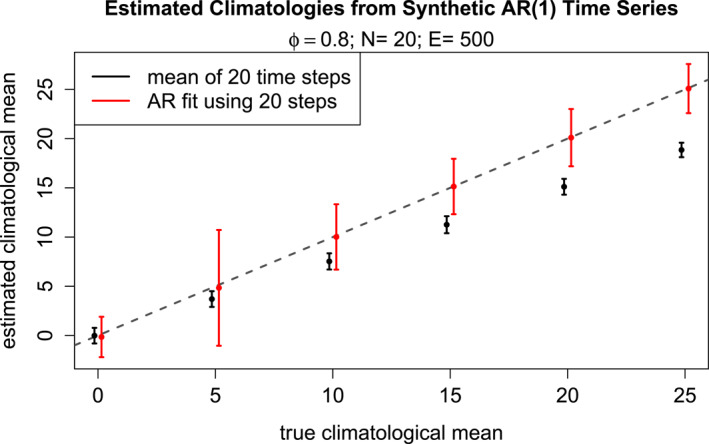
Climatologies estimated from synthetic time series generated by the AR(1) model $y_{t}=0.8y_{t-1}+k+w_{t}$ for different values of k and different realizations of $w_{t}$, each initialized at the same value $y_{1}=0$ and run to t=20. The climatologies are estimated by computing the average of 20 time steps (black), and by fitting the time series to an AR(1) model and then substituting the resulting parameters into Equation 2 (red). The dots and error bars show the mean and one standard deviation of the estimates over 500 realizations of synthetic time series.

Solutions of Equation 1 with $y_{1}=0$, ϕ=0.8 and $\sigma_{w}=1$ for selected values of k are shown in Figure 1. As can be seen, each solution “drifts” away from its initial value and toward its climatological mean 5k (since 1/(1−0.8)=5). This behavior is similar to the response of climate models to changing parameter values (as will be shown in Section 5). Furthermore, the residuals $y_{t}-0.8y_{t-1}$ equal $k+w_{t}$ and hence are stationary and white. In other words, a nonstationary time series containing drift may be transformed into stationary white time series, from which the underlying AR parameters (e.g., k and $\sigma_{w}^{2}$) may be estimated. In practice the parameters in the transformation are unknown, but they may be estimated from time series even in the presence of climate drift. To illustrate, we generated 500 realizations of the AR process for different values of k, each starting at $y_{1}=0$ and lasting 20 time steps, as illustrated in Figure 1. For each synthetic time series, we computed the 20‐step mean. Also, we used ordinary least squares to fit each time series to the AR model Equation 1 and then computed the corresponding climatological mean Equation 2 by substituting fitted values of k and ϕ. The results are shown in Figure 2. As expected, time means underestimate the true climatological mean, since the time series are still drifting to their new climatology. In contrast, the AR fit produces unbiased estimates of the true climatological mean.

To adapt the autoregressive process for use in Earth System Models (ESMs), several generalizations are necessary. First, the AR process must accommodate multiple variables, reflecting the variety of physical variables simulated by ESMs, such as surface temperature, precipitation, and top‐of‐atmosphere radiative fluxes. The generalization of the AR model to multiple variables is called a Vector Autoregressive Model, denoted VAR (Lütkepohl, 2005). If the number of variables is S, then in this generalization the state variable $y_{t}$ becomes an S‐dimensional vector and ϕ becomes an S×S matrix. Second, the AR process should account for non‐stationary variations such as the annual cycle and climate change trends. This is achieved in VAR models by integrating time‐dependent forcing functions, $f_{1}\left(t\right),\text{…},f_{J}\left(t\right)$, which may include periodic functions to simulate annual cycles, as well as time series of atmospheric composition or solar insolation for external forcings. The resulting model is called a Vector Autoregressive Model with Exogenous Variables and denoted VARX. Third, the AR process should account for the kinds of autocorrelation structure that might occur in climate time series. This can be done by including additional lags in the AR model.

The above generalizations lead to the following Vector Autoregressive Model with Exogenous Variables of order P, or VARX(P):

(3) $$y_{t}=\sum_{p=1}^{P}A_{p}y_{t-p}+\sum_{j=1}^{J}d_{j}f_{j}\left(t\right)+w_{t},$$

where t is time in months and

$$\begin{array}{lll} y_{t} & \in R^{S} & \text{the state vector at time}\;t\;(\text{in months})\\ A_{p} & \in R^{S\times S} & \text{matrix of AR parameters for}\;p=1,\text{…},P\\ f_{j}\left(t\right) & \in R^{1} & \text{forcing function at time}\;t\;\text{for}\;j=1,\text{…},J\\ d_{j} & \in R^{S} & \text{transfer function for the}\;j'\text{th}\;\text{forcing}\\ w_{t} & \in R^{S} & \text{Gaussian white noise with covariance matrix}\;\Gamma . \end{array}$$

The parameters $\left\{A_{1},\text{…},A_{p},d_{1},\text{…}d_{J},\Gamma \right\}$ in Equation 3 will be called *VARX parameters*. These parameters will be estimated from observations and ESM simulations. The parameters in the ESM will be denoted by the K‐dimensional vector θ and called *ESM parameters*. Note that the intercept is included by defining $f_{1}\left(t\right)=1$.

Although a VARX model can capture climate drift, one may still be concerned that it is too simplistic to emulate the statistics of an ESM. In fact, a special case of the VARX model, known as the Linear Inverse Model (LIM), can reproduce many statistical characteristics of the climate system, including various climate phenomena like the El Niño‐Southern Oscillation (ENSO), the Madden‐Julian Oscillation (MJO), and the Atlantic Multidecadal Variability (AMV) (Newman, 2007; Penland & Sardeshmukh, 1995; Vimont, 2012; Whitaker & Sardeshmukh, 1998; Zanna, 2012). Often, autocorrelation structures encountered in climate time series can be captured by a relatively low‐order autoregressive model. If the VARX model accurately captures the statistics of an ESM, then it is reasonable to assume that findings derived from the VARX model are applicable to the ESM.

The purpose of this paper is to develop a tuning strategy for ESMs that can account for climate drift. Our strategy for doing this is to develop a tuning algorithm based on the above VARX model. While numerous estimation methods have been proposed in the literature, most of these ignore climate drift. Another common feature of previous methods is that they involve additional parameters beyond those in the ESMs. Specifically, tuning problems fall into a class of problems called inverse problems, which tend to be ill‐posed. A standard approach to solving ill‐posed problems is to impose constraints on the parameters, or equivalently to regularize the problem (Hastie et al., 2009). Such constraints often can be interpreted in a Bayesian framework as specifying a prior distribution on the parameters. Unfortunately, the prior often is empirical and therefore involves additional parameters, often called hyperparameters, which also must be estimated. In this study, we opt for regularizing the problem by reducing the dimension of the state vector. Specifically, our state vector comprises S time series of sea surface temperature (SST) patterns. While other types of state vectors could be considered, and no claims are made as to whether SST is optimal, SST is used frequently at modeling centers for tuning ESMs (Hourdin et al., 2016). Choosing a low‐dimensional state vector is an implicit form of regularization. A virtue of this type of regularization is that it mitigates ill‐posedness while circumventing the need to explicitly specify a prior distribution on the parameters. Once the unregularized algorithm is formulated, it is straightforward to derive the associated regularized method from Bayes' theorem (as we will do in Appendix C).

The remainder of this paper is organized as follows. The next section introduces a generalized VARX model that includes dependencies on ESM parameters. The maximum likelihood estimates (MLEs) of the VARX parameters and the associated information matrix are given in Appendices A, B, C. This method is called VARX‐MLE‐F. It turns out that VARX‐MLE‐F performs poorly in small samples (as will be shown in Section 5). We hypothesize that the problem lies in the nonlinearity of the likelihood function. Accordingly, in Section 2, an alternative method, called VARX‐MLE‐L, is proposed in which the nonlinearity is removed by estimating parameters in two linear steps. Section 3 discusses alternative tuning strategies that ignore drift and can used as comparison. Section 4 describes the particular ESM used in this study and the state vector used for tuning and ESM parameters being tuned. Section 5 presents our results. The concluding section summarizes our results.

R codes for the above algorithms can be found at DelSole (2024b), and the ESM data used in the results section can be found at DelSole (2024a).

## 2 Tuning Strategies Based on VARX Models

Our tuning strategy assumes that all time series are realizations from a VARX model, and that the VARX model depends on the ESM parameters. Following standard practice, we focus on tuning the climatology. The climatology of the VARX model may be obtained from the fact that the general solution of a VARX model can be decomposed into two distinct parts: the homogeneous part and the inhomogeneous part. The inhomogeneous part satisfies the VARX model without random forcing $w_{t}$ and represents the model's response to external forcing; this is the climatology of the system. The climatology includes the long‐term mean and the seasonal cycle. In contrast, the homogeneous part satisfies the VARX model without deterministic forcing $\left\{d_{j}f_{j}\left(t\right)\right\}$ and defines the random internal variability of the system. The full solution is the sum of the homogeneous and inhomogeneous parts and can be interpreted as the sum of the internal variability plus the response to external forcing. In some contexts, the climatology might include long‐term trends due to external forcings, such as changes in atmospheric composition or solar insolation, but these trends are not a factor in our study (by design) and will be ignored.

To derive the climatology, we use the fact that any discrete periodic function can be represented by a discrete Fourier transform. Accordingly,

(4) $$\sum_{j=1}^{J}d_{j}f_{j}\left(t\right)=\sum_{h=1}^{H}d_{2h-1}\;\cos\left(\omega_{h}t\right)+\sum_{h=1}^{H}d_{2h}\;\sin\left(\omega_{h}t\right)+d_{2H+1},$$

where H is the number of annual harmonics, t is time in months, and $\omega_{h}=2\pi h/12$. Any annual cycle can be captured using the Nyquist frequency H=6. However, for $\omega_{6}$, the sine term equals zero and the cosine term is a triangle wave which, in practice, has negligible amplitude and can be excluded. In practice, then, the complete annual cycle can be captured using H=5, which corresponds to choosing 10 forcing functions (five for the cosine and five for the sine terms) and an intercept, giving J=11.

The response of the VARX model to the forcing function Equation 4 and with $w_{t}=0$ is the inhomogeneous solution to the VARX model. This solution can be obtained by temporarily working with complex variables. Specifically, if the forcing function is

$$f_{h}\left(t\right)=e^{i\omega_{h}t},$$

then the corresponding inhomogeneous solution is

(5) $$y_{t,h}^{\text{IN}}={\left(I-\sum_{p=1}^{P}A_{p}e^{-ip\omega_{h}t}\right)}^{-1}d_{h}.$$

Because the VARX model is linear, the principle of superposition applies, allowing us to solve for the response to each individual Fourier component separately, which can be added together to obtain the total response. Note that the constant climatological mean is included in the above solution as the special case $\omega_{j}=0$.

Solution Equation 5 shows that the climatological seasonal cycle of the VARX model depends on both the AR parameters $A_{1},\text{…},A_{p}$ and on the external forcing parameter $d_{j}$. In principle, both sets of parameters could depend on the ESM parameters. However, the inverse problem associated with this general case is complicated and is tantamount to tuning both the climatology and the internal variability. To simplify the problem, we assume the ESM parameters influence only the forcing parameters $\left\{d_{j}\right\}$, and that this influence is linear, yielding the parameterization

(6) $$d_{j}=c_{j}+C_{j}\theta \;\text{for}\;j=1,\text{…},J.$$

As can be seen from Equation 5, this parameterization implies that we are attempting to tune only the climatological mean and the annual cycle, leaving unchanged the statistics of the noise (modeled by Γ) and statistical dependencies in time (modeled by $A_{1},\text{…},A_{P}$). Although this assumption is restrictive, the parameterization Equation 6 is sufficiently general to allow the ESM parameters to affect any aspect of the climatology (e.g., amplitude, phase, frequency). To be clear, the solution Equation 5 is not needed for the tuning algorithms presented below, but was presented here to clarify the relation between the climatology and the parameters of the VARX model.

Substituting Equations 6 into Equation 3 yields the model

(7) $$y_{t}=\sum_{p=1}^{P}A_{i}y_{t-p}+\sum_{j=1}^{J}\left(c_{j}+C_{j}\theta \right)f_{j}\left(t\right)+w_{t}$$

where

$$C_{j}\in R^{S\times K}\;\text{and}\;c_{j}\in R^{S\times 1}.$$

The quantities $\left\{A_{j},c_{j},C_{j},\Gamma \right\}$ are *nuisance parameters*, in the sense that our goal is to estimate θ, but achieving this goal requires knowing the nuisance parameters. While it is possible that $A_{p}$ and Γ also could depend on θ, incorporating this dependence leads to a much more complex estimation problem involving an even larger number of nuisance parameters. As an example, assuming that $A_{p}$ depends linearly on θ leads to the parameterization $A_{p}=B_{0}+\theta_{1}B_{1}+\text{⋯}+\theta_{K}B_{K}$, which adds $S^{2}PK$ nuisance parameters, which grows quadratically with S. In principle, our framework could be used tune both climatology and internal variability, but this is a more ambitious problem that lies outside the scope of the present paper.

Our goal is to find the parameter values θ such that the statistics generated by the ESM are consistent with those observed in the real Earth system, to within observational uncertainties. To achieve this, two distinct data sets are available. The first is the observational data set $y_{t}^{o}$, where $t=1,2,\text{…},N_{O}$. We call this the *target data*. The target data is assumed to be a realization of the VARX model Equation 7 with $\theta =\theta_{o}$, which is unknown. The second data set are the outputs of the ESM for *known* parameter values $\theta_{1},\text{…},\theta_{E}$, where E denotes ensemble size. The respective outputs also are assumed be realizations from the VARX model Equation 7 with θ values $\theta_{1},\text{…},\theta_{E}$. We call these outputs the *perturbation runs* and denote them by $y_{t}^{\left(1\right)}\text{…},y_{t}^{\left(E\right)}$, with respective time lengths $N_{1},\text{…},N_{E}$, which may differ.

A natural approach to estimating $\theta_{o}$ is the maximum likelihood method. A basic reference that introduces VARX models and the maximum likelihood method for estimating its parameters is Lütkepohl (2005). However, the problem of estimating $\theta_{o}$ is non‐standard and requires a separate treatment. The maximum likelihood estimate (MLE) of $\theta_{o}$ and its associated covariance matrix is derived in Appendices A and B. Merely writing out the likelihood function requires an intricate notation, so we refer the reader to Appendix for further details about the MLE. Importantly, the estimate is obtained by solving a nonlinear system of equations. The nonlinearity arises from the fact that $\theta_{o}$ appears in VARX model only through the product $C_{j}\theta_{o}$, where $C_{j}$ is an unknown nuisance parameter. Unfortunately, results shown in Section 5 will demonstrate that the MLEs are inaccurate for small sample sizes. A regularized version of MLE also is derived in Appendix C by defining a prior distribution on $\theta_{o}$ and applying Bayes' theorem. However, the regularized version also performs poorly in our experiments, as will be discussed in more detail in Section 5.

We suspect that the poor performance of the MLEs from small samples is related to the nonlinearity mentioned above. An alternative method that avoids this nonlinearity is to separate the estimation of the nuisance parameters $\left\{A_{i},c_{j},C_{j},\Gamma \right\}$ from the estimation of $\theta_{o}$. For instance, the nuisance parameters $\left\{A_{i},c_{j},C_{j},\Gamma \right\}$ could be estimated using only the perturbation runs. In this case, the values of $\left\{\theta_{e}\right\}$ are known and the nuisance parameters $\left\{A_{i},c_{j},C_{j},\Gamma \right\}$ can be estimated by Ordinary Least Squares (OLS). Then, conditional on the estimates of $\left\{A_{i},c_{j},C_{j},\Gamma \right\}$, model Equation 7 has only one unknown, namely $\theta_{o}$, which can estimated by OLS from observational data. A shortcoming of this procedure is that no observational data is used to inform the nuisance parameters. An alternative two‐step procedure that incorporates more observational data is the following. First, estimate the nuisance parameters $\left\{A_{i},d_{j},c_{j},C_{j},\Gamma \right\}$ from the following equations

(8) $$\text{target}\;y_{t}^{o}=\sum_{p=1}^{P}A_{p}y_{t-p}^{o}+\sum_{j=1}^{J}d_{j}f_{j}\left(t\right)+w_{t}^{o}$$

(9) $$\text{perturbation}\;\text{runs}\;y_{t}^{e}=\sum_{i=p}^{P}A_{p}y_{t-p}^{e}+\sum_{j=1}^{J}c_{j}f_{j}\left(t\right)+\sum_{j=1}^{J}C_{j}\theta_{e}f_{j}\left(t\right)+w_{t}^{e},$$

where $w_{t}^{o}$ and $w_{t}^{e}$ are independent but have the same covariance matrix Γ. The nuisance parameters $\left\{A_{i},d_{j},c_{j},c_{j}\right\}$ appear linearly in the above models and therefore can be estimated by OLS. The residuals can then be used to estimate Γ. Importantly, this procedure utilizes both the target and perturbation runs to estimate $\left\{A_{i},d_{j},c_{j},c_{j},\Gamma \right\}$, which can lead to more accurate estimates compared to using only the perturbation runs. Let the resulting estimates be denoted $\left\{{\tilde{A}}_{i},{\tilde{d}}_{j},{\tilde{c}}_{j},{\tilde{c}}_{j},\tilde{\Gamma }\right\}$. Then, conditional on these estimates, $\theta_{o}$ is estimated from the model

$$\sum_{j}{\tilde{d}}_{j}f_{j}\left(t\right)=\sum_{j}\left({\tilde{c}}_{j}+{\tilde{C}}_{j}\theta_{o}\right)f_{j}\left(t\right)+\epsilon ,$$

where $cov\left[\epsilon \right]=\tilde{\Gamma }$. The maximum a posteriori estimate is obtained by minimizing

(10) $$\sum_{t}{\tilde{e}}^{T}\left(t\right){\tilde{\Gamma }}^{-1}\tilde{e}\left(t\right),$$

where superscript T denotes the transpose operation and

$$\tilde{e}\left(t\right)=\sum_{j}\left({\tilde{d}}_{j}-{\tilde{c}}_{j}-{\tilde{C}}_{j}\theta_{o}\right)f_{j}\left(t\right).$$

The minimum of Equation 10 is achieved at

(11) $${\tilde{\theta }}_{o}={\tilde{U}}^{-1}\tilde{v},$$

where

(12) $$\tilde{U}=\sum_{j}\sum_{j^{\prime }}\left(f_{j}^{T}f_{j^{\prime }}\right){\tilde{C}}_{j}^{T}{\tilde{\Gamma }}^{-1}{\tilde{C}}_{j^{\prime }}$$

(13) $$\tilde{v}=\sum_{j}\sum_{j^{\prime }}\left(f_{j}^{T}f_{j^{\prime }}\right){\tilde{C}}_{j}^{T}{\tilde{\Gamma }}^{-1}\left({\tilde{d}}_{j^{\prime }}-{\tilde{c}}_{j^{\prime }}\right).$$

Note that here the estimation of $\theta_{o}$ is a linear problem, in contrast to the full MLE solution. Nevertheless, the above solution is closely related to the MLE derived in Appendix A (For instance, the counterpart to Equation 11 is Equation A12). The associated covariance matrix follows from the information matrix associated with Equation 10, namely

(14) $$cov\left[{\tilde{\theta }}_{o}\right]={\tilde{U}}^{-1}.$$

We call ${\tilde{\theta }}_{o}$ in Equation 11 the *VARX‐MLE‐L* estimate, while the full, nonlinear MLE described in Appendix A will be called the *VARX‐MLE‐F*.

## 3 Tuning Strategies That Ignore Climate Drift

To place our tuning algorithm into perspective, we compare it to other algorithms that ignore climate drift. Accordingly, only first‐order moments are considered in this section. Thus, the ESM is run using the values $\theta_{1},\text{…},\theta_{E}$, and the corresponding time means are denoted ${\bar{y}}_{1},\text{…},{\bar{y}}_{E}$. The observed time mean is denoted ${\bar{y}}^{o}$.

Our first algorithm is based on the Kalman Filter along the lines proposed by Schneider et al. (2017). This algorithm applies the Kalman Filter to the state vector

$$z=\left(\begin{array}{c} \bar{y}\\ \theta \end{array}\right),$$

and assumes that observations come from the model

(15) $${\bar{y}}^{o}=Hz+r,$$

where r is a random vector with zero mean and covariance matrix $r^{2}R$ (specified below). Typically, the time means are observed directly while θ is unobserved, hence

(16) $$H=\left[I_{S\times S}\;\;0_{S\times K}\right],$$

so that ${\bar{y}}^{o}=\bar{y}+r$.

The prior distribution of z is assumed to be normal with mean $\mu_{B}$ and covariance matrix $\Sigma_{B}$, where B stands for “background.” These parameters are estimated from the perturbation runs as

$$\mu^{B}=\frac{1}{E}\sum_{e=1}^{E}\left(\begin{array}{c} {\bar{y}}^{e}\\ \theta_{e} \end{array}\right),$$

and

$$\Sigma^{B}=\frac{1}{E-1}\sum_{e=1}^{E}\left(\left(\begin{array}{c} {\bar{y}}^{e}\\ \theta_{e} \end{array}\right)-\mu_{B}\right){\left(\left(\begin{array}{c} {\bar{y}}^{e}\\ \theta_{e} \end{array}\right)-\mu_{B}\right)}^{T}.$$

Then, the Kalman Filter produces a posterior distribution, called the “analysis,” that is normal with mean and covariance matrix

(17) $$\mu^{A}=\mu^{B}+\Sigma^{B}H^{T}{\left(H\Sigma^{B}H^{T}+r^{2}R\right)}^{-1}\left({\bar{y}}^{o}-H\mu^{B}\right)$$

(18) $$\Sigma^{A}=\Sigma^{B}-\Sigma^{B}H^{T}{\left(H\Sigma^{B}H^{T}+r^{2}R\right)}^{-1}H\Sigma^{B}.$$

Up to this point, all parameters except r and R have been specified. Schneider et al. (2017) suggest using a diagonal matrix for R, with diagonal elements equal to the climatological variances of the elements of ${\bar{y}}^{o}$. The hyperparameter r then is chosen to optimize performance in idealized experiments, but no procedure for selecting this parameter in practical situations was proposed. This situation is closely related to a procedure called covariance inflation (Anderson, 2007). Specifically, covariance inflation involves replacing the background covariance matrix $\Sigma_{B}$ with a re‐scaled version $\gamma \Sigma_{B}$, where γ is a scaling factor. It is simple to show that

$$\gamma \Sigma^{B}H^{T}{\left(\gamma H\Sigma^{B}H^{T}+R\right)}^{-1}=\Sigma^{B}H^{T}{\left(H\Sigma^{B}H^{T}+\frac{1}{\gamma }R\right)}^{-1},$$

which shows that re‐scaling $\Sigma_{B}$ is equivalent to re‐scaling R in Equation 17. Given this connection, one could apply covariance inflation techniques to specify r. Various algorithms for adaptively choosing this inflation factor have been proposed (Anderson, 2007; El Gharamti, 2018; Iglesias & Yang, 2021; Li et al., 2009). Here, we follow an approach proposed by Lydeen et al. (2024), who showed that the above algorithm can be formulated equivalently as a ridge regression problem, where r plays the role of the ridge parameter. Then, standard cross‐validation techniques can be used to select r, thereby eliminating the need for additional experiments to select r. The resulting algorithm is fully specified with no tunable hyperparameters. We call this algorithm *KalmRidge*.

If the means and covariances of $y_{t}^{e}$ and $y_{t}^{o}$ are computed from time series of the same length, and the time series are sufficiently long, then KalmRidge should yield reasonable assessments of uncertainty. However, the requirement that the target time series and the perturbation runs be the same length is quite restrictive. For instance, observational time series can be quite long, say 50–100 years, while the perturbation runs are typically much shorter. As a result, KalmRidge cannot incorporate most of the observational time series, leading to a waste of information.

An algorithm that allows different lengths of time series is the following. Bayes theorem gives the posterior distribution of the parameters as

$$p\left(\theta |\left\{y_{t}\right\}\right)=p\left(\left\{y_{t}\right\}|\theta \right)p\left(\theta \right)/p\left(\left\{y_{t}\right\}\right),$$

where $\left\{y_{t}\right\}$ denotes the full time series (see Equation A1 for more details). Ignoring autocorrelations, $p\left(\left\{y_{t}\right\}|\theta \right)$ for normal distributions is equivalent to the model

(19) $$y_{t}=L\theta +\mu_{y|\theta }+\epsilon_{t}\;\text{where}\;\epsilon_{t}\overset{IID}{∼}N\left(0,\Sigma_{Y|\theta }\right).$$

The parameters L and $\mu_{y|\theta }$ are merely the multivariate extension of the slope and intercept of a regression line. Although this model ignores autocorrelation in $y_{t}$, it has the advantage that statistical moments can be expressed in terms of the model parameters. Specifically, since $\epsilon_{t}$ in Equation 19 is white noise, sample means $\bar{y}$ computed over N time steps can be treated as data generated by the model

$$\bar{y}=L\theta +\mu_{y|\theta }+\bar{\epsilon }\;\text{where}\;\bar{\epsilon }\overset{IID}{∼}N\left(0,\Sigma_{Y|\theta }/N\right).$$

Importantly, the parameters $\left\{L,\Sigma_{Y|\theta },\mu_{y|\theta }\right\}$ can be estimated by ordinary least squares, even if the perturbation runs may have different lengths.

In principle, we could include a normal prior for p(θ) and derive a posterior, but the resulting algorithm would depend on the hyperparameters associated with the prior. To avoid such hyperparameters, we instead assume an uninformative prior, and then compute the maximum a posteriori (MAP) estimate by finding the parameters that maximizes $p\left(\theta |\left\{y_{t}\right\}\right)$. This is equivalent to a Generalized Least Squares problem and yields the mean and covariance

(20) $$\mu_{\text{cGLS}}={\left(L^{T}\Sigma_{Y|\theta }^{-1}L\right)}^{-1}L^{T}\Sigma_{Y|\theta }^{-1}\left({\bar{y}}_{0}-\mu_{y|\theta }\right)$$

(21) $$\Sigma_{\text{cGLS}}={\left(L^{T}\Sigma_{Y|\theta }^{-1}L\right)}^{-1}/N_{o}.$$

This algorithm will be called conditional Generalized Least Squares (cGLS), since it is conditioned on $\left\{L,\Sigma_{Y|\theta },\mu_{y|\theta }\right\}$. In contrast to the MLE method discussed in Appendix A, the cGLS algorithm involves no nonlinear optimization. Instead, it only involves two successive least squares problems: first the parameters in Equation 19 are estimated using only perturbation runs, then the resulting parameters are substituted into Equations 20 and 21 to estimate the distribution parameters of $p\left(\theta |\left\{y_{t}\right\}\right)$.

While the cGLS algorithm can assimilate perturbation runs and observations of arbitrary lengths, it assumes independent samples and therefore ignores autocorrelation in $y_{t}$. Also, the cGLS algorithm is conditional on estimates of parameters $\left\{L,\Sigma_{Y|\theta },\mu_{y|\theta }\right\}$, but the uncertainty of these estimates are not taken into account in the final uncertainty. For these reasons, we anticipate that cGLS will systematically underestimate the true uncertainty.

## 4 The Climate Model and Data

The climate model chosen for this study is the Community Earth System Model version 2 (CESM2). This model is discussed in detail in Danabasoglu et al. (2020) and will be summarized only briefly here. CESM2 is a comprehensive Earth System Model with prognostic models for the atmosphere, ocean, land, land ice, river runoff, and sea ice. As such, it simulates at least 1 million variables. The number of tunable parameters is large—the namelist for the atmospheric model (CAM6.3) alone contains 1,065 parameters. Moreover, CESM2 is computationally expensive to run and typically is run only on state‐of‐the‐art supercomputers.

For the experiments discussed in this paper, we use one run as the “target” and runs with different parameter values as the perturbation runs. In this setup, there exists a value of the ESM parameters that perfectly matches the model that generated the target, which is unrealistic. Nevertheless, this experimental set up was chosen because it allows us to assess the accuracy of the estimated parameters, an assessment that could not be done if real observations were used

As a first test case, two model parameters are chosen, so K=2. The particular parameters were chosen because they have been the subject of tuning experiments in previous studies and have emerged as key tuning parameters (Guo et al., 2014; Woelfle et al., 2019). The first is a parameter called CLUBBGamma (γ) in the subgrid parameterization of CESM2 (Larson, 2020). This is a non‐dimensional parameter that influences the tails of the distribution for the subgrid vertical velocity. Specifically, larger values imply longer tails in the velocity, which lead to stronger entrainment at the top of the planetary boundary layer, which reduces the amount and thickness of low clouds. The second is a parameter called DCS in the microphysical parameterization in CESM2 (Woelfle et al., 2019). DCS is the diameter (in units of μm) to which a cloud ice particle must grow before it is converted to snow and subsequently precipitated out of the cloud at a particular model level. Use of a larger DCS value enables the retention of larger ice particles within glaciated clouds.

**Figure 3:**
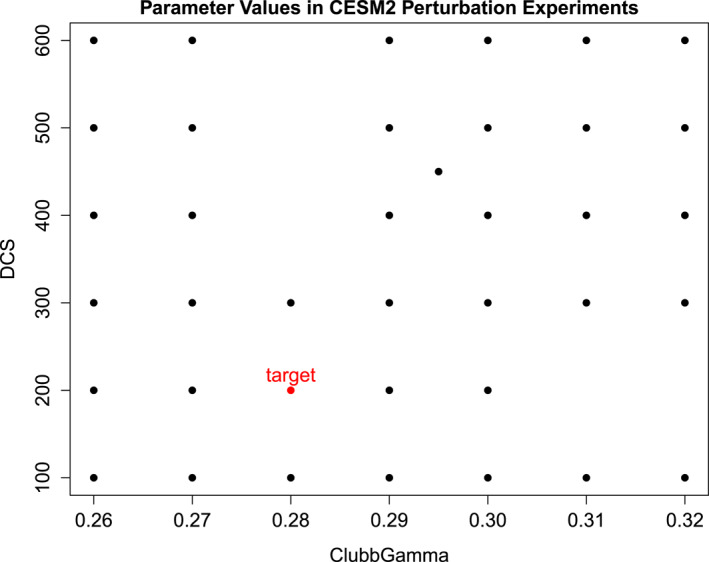
Parameter values chosen for CLUBBGamma (dimensionless, *x*‐axis) and DCS (μm, *y*‐axis) in the CESM2 perturbation experiments (black dots), and the target value (red dot).

The target value of CLUBBGamma is 0.28 and the target value of DCS is 200 μm. A 500‐year run with these target values is available. In addition, 37 10‐year runs were made with different choices of this pair of parameter values. These runs are the perturbation runs. The precise parameter values are indicated in Figure 3.

**Figure 4:**
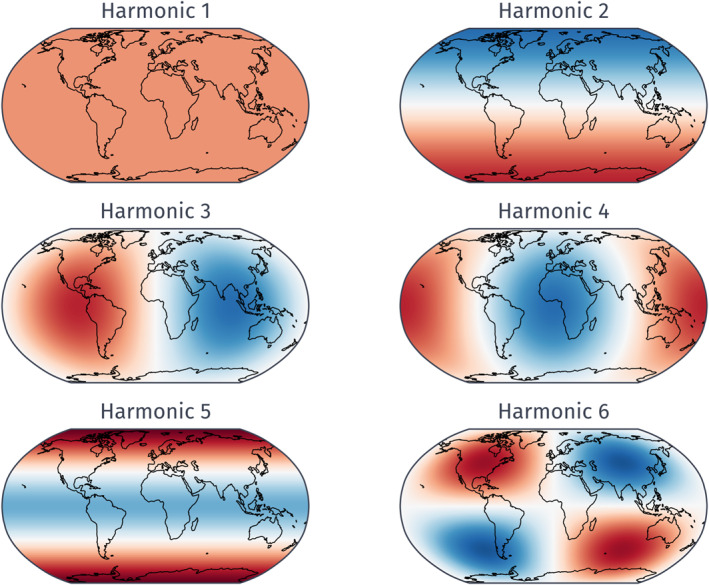
The first six spherical harmonics on a globe. Courtesy of Nikki Lydeen.

Following previous tuning studies, we analyze sea surface temperature (SST). To isolate large‐scale features, we project simulated SSTs onto the leading spherical harmonics, masking out land. The first six spherical harmonics are shown in Figure 4 for reference. Projecting SST onto the first harmonic corresponds to computing the spatial average temperature over the ocean.

## 5 Results

We analyze monthly means of CESM2 time series. Although 500 years of a control simulation are available, we limit the target data size $N_{o}$ to 50 years to be comparable to observational data sets that might be used for model tuning. In our experiments, each perturbation run is the same length, so $N_{e}$ is the same for all ensemble members e. We have explored a wide range of values of $S,P,H,E,N_{e}$, but only a representative subset of these results will be presented here.

**Figure 5:**
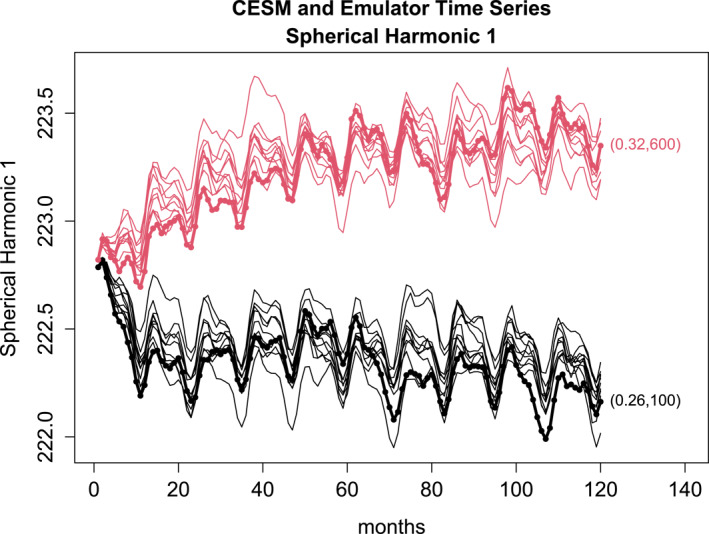
Projection of CESM2 runs onto the first spherical harmonic (thick line with dots), and 10 realizations of the VARX model starting from the same initial condition (thin lines). The VARX model is fit using H=5,P=1,S=20. CESM runs start with the same initial condition. Runs with different values of CLUBBGamma and DCS are distinguished by different colors (numerical values indicated at end of the time series).

To support the reasonableness of the VARX model, we first use only perturbation runs to fit the VARX model to construct an *emulator* for the ESM. This fitting problem leads to an ordinary least squares problem since the parameter values $\theta_{1},\text{…},\theta_{E}$ are known. Then, the emulator is applied to generate synthetic time series. The resulting time series are compared to independent realizations of the ESM to judge the reasonableness of the VARX model. For the first illustration, we choose 20 spherical harmonics, thus S=20, and P=1 and H=5. Time series of the first spherical harmonic for CESM and for 10 realizations of the VARX model are shown in Figure 5. As can be seen, CESM2 drifts to new climatologies, and the VARX model captures the overall drift and variability over the 10‐year period. These results suggest that the VARX model is a reasonable emulator for the CESM.

**Figure 6:**
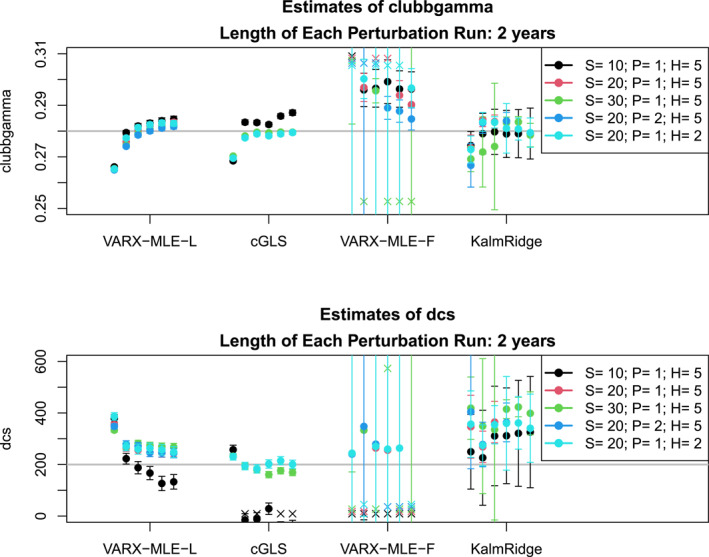
Estimates of the CESM parameters CLUBBGamma (top) and DCS (bottom) for different estimation methods (*x*‐axis) and different configurations of the perturbation runs and VARX model (colors). S is the number of spherical harmonics; P is the order of the VARX model; H is the number of harmonics. The error bar shows two standard errors of each estimate. The results for ensemble sizes {10,15,20,25,30,35} are displayed from left to right for each method. All cases use a target time series of 50 years and perturbation runs of length 2 years. A cross “×” indicates an estimate beyond the upper or lower range of the displayed *y*‐axis.

A key question is if ESM parameters can be estimated using perturbation runs during the drift phase. To address this question, we fix the maximum length of the perturbation runs to 2 years, which is within the drifting phase displayed in Figure 5. The estimated ESM parameters for different configurations of the perturbation runs and VARX models are shown in Figure 6. The associated confidence intervals are obtained as explained earlier (e.g., Equation 14 for VARX‐MLE‐L; Equation B8 for VARX‐MLE‐F; Equation 18 for KalmRidge; Equation 21 for cGLS). As can be seen, the VARX‐MLE‐L and cGLS methods produce reasonable estimates, provided 20 or more spherical harmonics are used and the ensemble size is 15 or more. As anticipated, the confidence intervals are too narrow and usually do not include the true value. In contrast, the VARX‐MLE‐F not only produces less accurate estimates, but many of the DCS estimates are negative and therefore unphysical. KalmRidge produces less accurate estimates than VARX‐MLE‐L and cGLS, but the confidence intervals are wider and include the true parameter. The VARX‐MLE‐L and cGLS are remarkably insensitive to different choices of S,P,H. We remind the reader that the performance of KalmRidge may not be representative because we have restricted the dimension S of the state vector to relatively small values.

We now examine the performance of individual methods more closely. Since the goal is to estimate parameters with the least possible computational expense, we present our results in terms of the computational effort. Specifically, we present results in terms of the combined length of perturbation runs, $EN_{e}$. We explored the choices $E=\left\{10,15,20,25,30,35\right\},N_{e}=\left\{1,2,3,4,5,10\right\}$ years, S={10,20,30}, P={1,2} and H={2,5}. Note that the same combined length of perturbation runs $EN_{e}$ may correspond to different run lengths (for instance, 10×3 and 15×2 both equal 30). As a reminder, the other parameters are K=2, J=11, and $N_{o}=50*12=600$.

**Figure 7:**
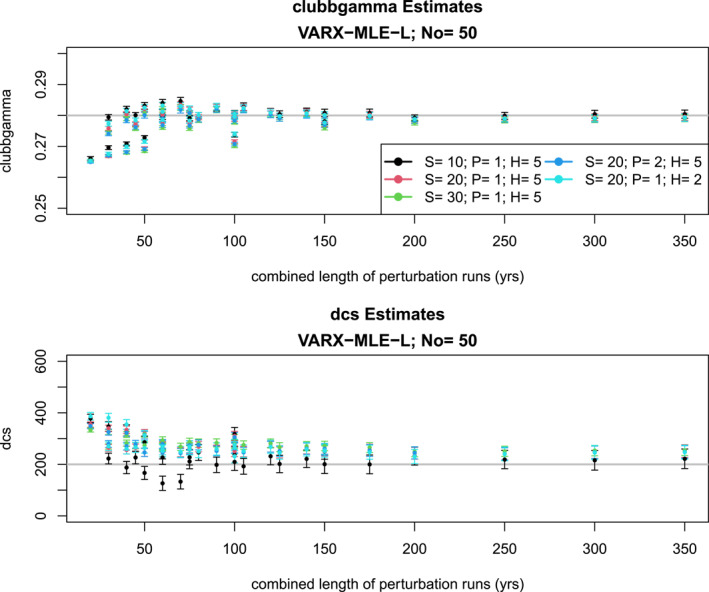
The VARX‐MLE‐Ls (dots) of the CESM parameters CLUBBGamma (top) and DCS (bottom) for various configurations of the perturbation runs and VARX model. S is the number of spherical harmonics; P is the order of the VARX model; H is the number of harmonics. Each estimate is plotted as a function of the combined length of the perturbation run $EN_{e}$. All cases use a target time series of 50 years. The error bar shows two standard errors of each estimate. A cross “×” indicates an estimate beyond the displayed upper or lower range of the *y*‐axis.

A representative sample of the VARX‐MLE‐Ls are shown in Figure 7. The VARX‐MLE‐Ls converge after about 100 years of combined length of perturbation runs, assuming the use of 20 or more spherical harmonics (i.e., S≥20). This result reflects a pattern that will be seen in other examples: the estimates converge when S≥20 and the combined length of perturbation runs exceeds 100 years. Under these conditions, the accuracy of the estimates remains nearly unchanged regardless of the ensemble size, the duration of individual perturbation runs, the order of the VARX model, or the number of harmonics employed.

However, the converged value is slightly too large for DCS. Also, the true value is outside two standard errors in many cases even for long perturbation runs. The fact that the uncertainty estimates of the VARX‐MLE‐Ls are overly confident was anticipated, since the algorithm ignores uncertainty in the estimates of the nuisance parameters.

**Figure 8:**
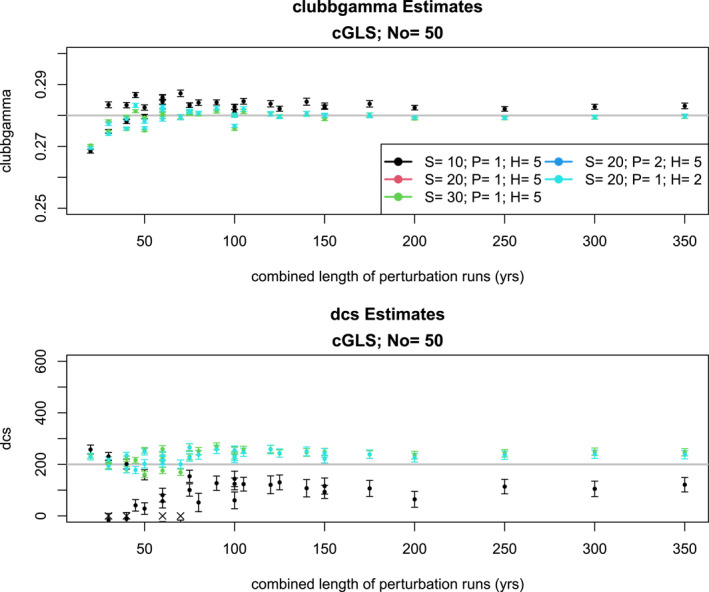
Same as Figure 7, but for conditional Generalized Least Squares.

Estimates from cGLS, shown in Figure 8, are similar to the VARX‐MLE‐Ls, including the overly narrow confidence intervals and the fact that the estimates converge after about 100 years of combined length of perturbation runs. Nevertheless, even for long effective perturbation runs, cGLS estimates do not converge to the true value. This bias is presumably due to the fact that cGLS ignores autocorrelation and drift.

**Figure 9:**
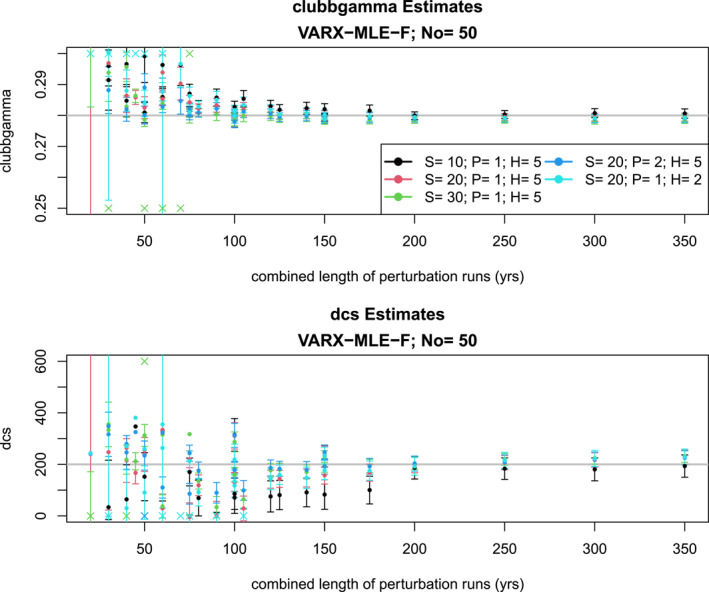
Same as Figure 7, but for the VARX‐MLE‐Fs.

Estimates from VARX‐MLE‐F are shown in Figure 9. In general, estimates of CLUBBGamma become independent of the combined length of perturbation runs after about 125 years, whereas DCS requires about 200 years. For sufficiently long perturbation runs (e.g., 200 or more years), the true value is within two standard errors of the maximum likelihood estimate. These results demonstrate the success of the method for sufficiently large samples. However, for combined length of perturbation runs less than 100 years, estimates of DCS are negative, which are not physical. Moreover, the uncertainty interval does not include the true parameter value for most cases under 100 years of perturbation runs.

**Figure 10:**
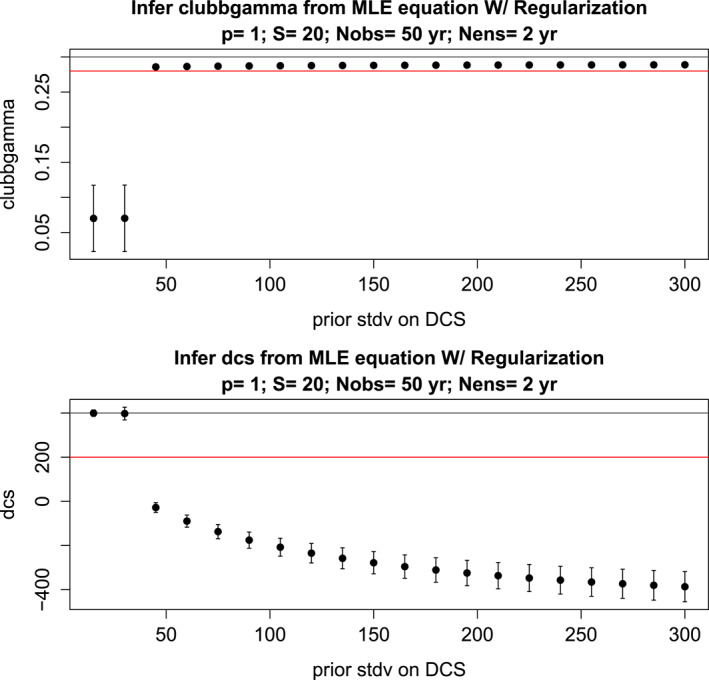
Results of regularized maximum likelihood estimation (dots) of the CESM parameters CLUBBGamma (top) and DCS (bottom) as a function of the standard deviation of the prior on DCS. The error bar shows two standard errors of each estimate. These results correspond to the following choices: S=20 is the number of spherical harmonics; P=1 is the order of the VARX model; H=5 is the number of harmonics; E=37 is the number of ensemble members; $N_{e}=$ 2 years is the length of the individual perturbation runs; the target time series of 50 years. The gray line shows the prior value and the red line shows the target value.

Since VARX‐MLE‐F method seems to fail at the shortest sample sizes, a regularized algorithm might produce better estimates in this small‐sample regime. Although we have examined numerous cases, we present only one representative case in detail, namely the case $P=1,S=20,E=37,N_{e}=2$ yrs, $N_{o}=$ 50 years, which produced the estimate γ=0.29 and DCS = −426. These are poor estimates of the target γ=0.28 and DCS = 200. For the prior, we assume the covariance matrix $\Sigma_{\theta \theta }$ is diagonal with diagonal elements $\sigma_{\gamma }^{2}$ and $\sigma_{\text{DCS}}^{2}$, which are the respective variances of γ and DCS in the prior distribution. We choose $\sigma_{\gamma }=0.1$, and allow $\sigma_{\text{DCS}}$ to vary. The prior mean is $\mu_{\gamma }=0.3$ and $\mu_{\text{DCS}}=400$. The regularized estimates as a function of $\sigma_{\text{DCS}}$ is shown in Figure 10. As can be seen, the regularization fails to give reasonable values of DCS—the regularized estimates are either strongly constrained to the prior mean, or abruptly drop to negative values over a narrow range of regularization parameter. The abrupt transition reflects the nonlinearity involved in the VARX‐MLE‐F.

The fact that maximum likelihood methods give unrealistically narrow uncertainty estimates for the smallest sample sizes presumably reflects the fact that these sample sizes are not sufficiently large for the asymptotic theory to be appropriate. It is possible that different choices of parameters for the prior may yield improved results, but this requires searching over parameter space. Moreover, in practice, the true parameter value is unknown, so some criterion that can be evaluated without knowing the truth needs to be used to specify the prior.

**Figure 11:**
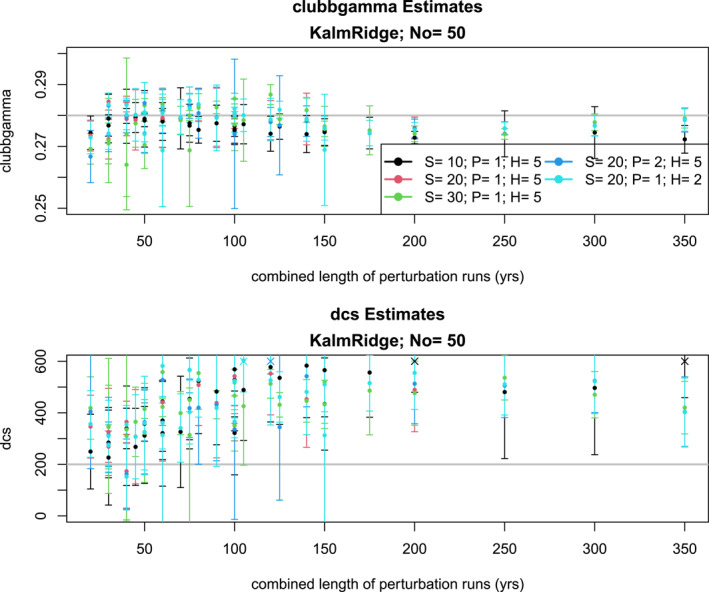
Same as Figure 7, but for KalmRidge.

A different approach to regularization is the KalmRidge algorithm. Here, the regularization parameter $r^{2}$ controls the degree to which the algorithm fits to the target data. Results from the KalmRidge algorithm are shown in Figure 11. As can be seen, these estimates have wider uncertainty intervals and much less accuracy that the VARX‐MLE‐L, which is not surprising in view of the restriction that the target time series must be the same length as the perturbation runs. Also, KalmRidge estimates do not converge to the true values for large combined length of perturbation runs. Our use of the KalmRidge algorithm appears less effective in this paper compared to our previous publication Lydeen et al. (2024). The key factor is that the current study imposes stricter constraints on the state space dimension, mainly due to the absence of regularization in our method. Specifically, this study applied KalmRidge to state space dimensions of approximately 20, whereas Lydeen et al. (2024) explored dimensions of one hundred or larger.

## 6 Summary and Conclusion

This paper presented algorithms for tuning Earth System Models using perturbation runs that have not reached statistical equilibrium. The basic idea is to assume time series come from VARX models. The advantage of VARX models is that their parameters can be estimated even when the time series exhibit climate drift. In principle, all parameters in the VARX model could depend on the ESM parameters, but for simplicity we assume only the exogeneous forcing terms depend on ESM parameters, which is tantamount to tuning only the climatology (including the annual cycle). The associated maximum likelihood estimate of the VARX parameters, as well as the associated information matrix and regularized version, are derived and presented in Appendices A, B, C. The associated likelihood function contains terms that depend nonlinearly on the VARX parameters and therefore require iterative methods to obtain maximum likelihood estimates. We called these estimates the VARX‐MLE‐Fs.

This algorithm was applied to estimate two parameters in the CESM2 model. Instead of targeting real observations, we target an independent run from CESM2 with known parameter values, which enables us to quantify the accuracy of the estimate relative to the target value. The time series were monthly means of sea surface temperature projected onto the leading spherical harmonics, masking out land.

Unfortunately, applying the VARX‐MLE‐F to these simulations resulted in poor estimates of the ESM parameters for small sample sizes. Specifically, for combined length of perturbation runs less than 100 years, the estimate often differed from the truth by more than a factor of two, and the true value often did not lie within two standard errors of the estimate. In fact, one of the parameters was estimated to be negative, which is unphysical. A regularized version of this algorithm failed to improve estimates in this small‐sample regime. These results are noteworthy because the maximum likelihood estimation method holds a unique status among various estimation techniques due to its desirable properties, such as consistency, asymptotic efficiency, and asymptotic normality under certain conditions. Our results demonstrate that the MLE method, even when regularized, performs poorly when the total length of perturbation runs is less than 200 years. It is particularly surprising that the regularized version was no better than the unregularized version. On the other hand, when the combined length of perturbation runs exceeded 150–200 years, VARX‐MLE‐Fs were within 1% of the true value and their confidence intervals included the true value, confirming that the VARX‐MLE‐F algorithm performs reasonably in large sample sizes.

We hypothesized that the small‐sample problems of VARX‐MLE‐Fs were related to the nonlinearity of the likelihood function. Accordingly, we proposed an alternative method, called VARX‐MLE‐L, that separated the estimation of nuisance parameters from estimation of the ESM parameters, resulting in a likelihood function that is linear in each estimation step. This method produced much more accurate estimates of ESM parameters even for 2‐year perturbation runs, provided 20 or more spherical harmonics were included in the VARX model. The results seem to support a simple rule of thumb: the VARX‐MLE‐L estimates converge provided the combined length of perturbation runs exceeds 100–200 years. Otherwise, the estimates were remarkably insensitive to different configurations of the VARX model (e.g., the number of annual harmonics, the number of spherical harmonics, or the order of the AR process) and to different ensemble sizes and perturbation run lengths. However, VARX‐MLE‐Ls did not converge to the true values for sufficiently long perturbation runs, and in many cases were too confident, presumably because the full information matrix was not used.

The question arises as to how well VARX‐MLE‐L compares to alternative methods that do not account for climate drift. One such algorithm is the KalmRidge algorithm proposed by Lydeen et al. (2024). In our study, KalmRidge produced less accurate estimates than VARX‐MLE‐L. One reason for this is that the dimension of the state space was restricted to about 20, for comparison purposes, whereas Lydeen et al. (2024) apply it to higher dimensions (100 or more). Another reason is that KalmRidge requires the target and perturbation runs to be of the same length. To circumvent this limitation, we proposed a new algorithm called conditional generalized least squares (cGLS). The cGLS algorithm produces much more accurate estimates than KalmRidge, with its performance being comparable to VARX‐MLE‐L. However, like VARX‐MLE‐L, cGLS failed to converge to the true values for long perturbation runs and exhibited overconfidence in its estimates.

It is noteworthy that cGLS, initially proposed as a benchmark, performs quite well even though it does not consider autocorrelation and drift. Further evaluation of this method is certainly warranted. Nevertheless, we maintain that methods that explicitly account for drift, such as VARX‐MLE‐F, offer a more promising basis for model tuning, despite their less‐than‐satisfactory performance here. We attribute the suboptimal performance of these methods primarily to the nonlinearity of the likelihood function when applied to small sample sizes. Further investigation into this nonlinearity could clarify its characteristics and motivate better approaches.

Among the assumptions made in our algorithm, the one that is most questionable is probably not the VARX model itself, but rather the assumption of linearity between ESM parameters and outputs, as embodied in Equation 6. If this assumption holds true, then reliable parameter estimates can be obtained in a single estimation step, especially with a large ensemble of perturbation runs. However, if the relation between parameters and outputs is nonlinear, it still may be possible to obtain reasonable estimates by iterating the algorithm. This iteration would involve refining the parameter estimates through successive perturbation runs, a process that, while potentially leading to more accurate results, also increases computational demands. Therefore, the choice between a single‐step or iterative approach needs to consider the trade‐off between computational costs and the potential gains in parameter accuracy.

A natural question in model tuning is whether the best fit parameters yield an ESM that is consistent with observations. In the experiments discussed here, there exists a value of the ESM parameters that will yield the data generating model. In reality, the ESM contains structural errors and hence no value of the ESM parameters will yield the data generating model. Happily, our algorithm fits naturally into a framework for deciding if the ESM is statistically distinguishable from observations. Specifically, a procedure for comparing VARX models, as proposed by DelSole and Tippett (2024), can be adapted for the tuning algorithm presented here. This procedure involves fitting both the target and perturbation runs to the VARX models Equations 8 and 9, but allowing the nuisance parameters to differ between the models. Then, the likelihood ratio method can be applied to test the hypothesis that the nuisance parameters of one VARX model equals those of the other. If this hypothesis cannot be rejected, then observations and the ESM have consistent internal variability and climatology, indicating that no further tuning is necessary.

A persistent deficiency of most of the above methods is that the confidence intervals are too narrow. Previous studies have noted this deficiency and proposed methods to overcome it (Cleary et al., 2021). It is conceivable that bootstrap methods also could be applied to advantage here.

## Data Availability

The codes and data used in this paper can be found at https://zenodo.org/doi/10.5281/zenodo.11260284.

## Appendix A Maximum Likelihood Estimate

This Appendix derives the maximum likelihood estimate of $\theta_{o}$ of the VARX model Equation 7. Our data set consists of a target data set $y_{t}^{o}$ where $t=1,\text{…},N_{O}$, and a set of perturbation runs from the ESM using parameter values $\theta_{1},\text{…},\theta_{E}$, whose respective outputs are $y_{t}^{\left(1\right)},\text{…},y_{t}^{\left(E\right)}$ with respective time lengths $N_{1},\text{…},N_{E}$.

It proves convenient to define the following matrix:

(A1)
$$\left\{y_{t}^{o}\right\}=\left(\begin{array}{c} {\left(y_{P+1}^{o}\right)}^{T}\\ {\left(y_{P+2}^{o}\right)}^{T}\\ \text{⋮}\\ {\left(y_{N_{O}}^{o}\right)}^{T} \end{array}\right)\in R^{\left(N_{o}-P\right)\times S}.$$

As indicated above, $\left\{y_{t}^{o}\right\}$ is a TIME × SPACE matrix in which the vector time series in question is expanded starting at t=P+1 and ending at the maximum value of t appropriate for that variable. By this definition,

$$\left\{y_{t-1}^{e}\right\}=\left(\begin{array}{c} {\left(y_{P}^{e}\right)}^{T}\\ {\left(y_{P+1}^{e}\right)}^{T}\\ \text{⋮}\\ {\left(y_{N_{E}-1}^{e}\right)}^{T} \end{array}\right)\;\text{and}\;\left\{y_{t-P}^{e}\right\}=\left(\begin{array}{c} {\left(y_{1}^{e}\right)}^{T}\\ {\left(y_{2}^{e}\right)}^{T}\\ \text{⋮}\\ {\left(y_{N_{E}-P}^{e}\right)}^{T} \end{array}\right)$$

Also, we define the TIME × 1 vectors

$$f_{j}^{o}=\left\{f_{j}^{o}\left(t\right)\right\}\;\text{and}\;f_{j}^{\left(e\right)}=\left\{f_{j}^{e}\left(t\right)\right\}$$

The likelihood of model Equation 7 is

(A2)
$$L=\left(\frac{L_{P}}{{\left(2\pi \right)}^{NS/2}|\Gamma {|}^{N/2}}\right)e^{-tr\left[\Gamma^{-1}Z\right]/2},$$

where $L_{P}$ are terms that depend on the first P values of the process,

(A3)
$$Z={\left(y-XB\right)}^{T}\left(y-XB\right),$$

where

$$y=\left(\begin{array}{c} y_{O}\\ y_{E} \end{array}\right),\;X=\left(\begin{array}{cc} X_{O1} & X_{O2}\\ X_{E1} & X_{E2} \end{array}\right)\;\text{and}\;B=\left(\begin{array}{c} B_{1}\\ B_{2} \end{array}\right),$$

and

$$\begin{array}{ll} y_{O} & =\left\{y_{t}^{o}\right\}\\ X_{O1} & =\left(\begin{array}{cccccc} \left\{y_{t-1}^{o}\right\} & \text{…} & \left\{y_{t-P}^{o}\right\} & f_{1}^{o} & \text{…} & f_{J}^{o} \end{array}\right)\\ X_{O2} & =\left(\begin{array}{ccc} f_{1}^{o}\theta_{o}^{T} & \text{…} & f_{J}^{o}\theta_{o}^{T} \end{array}\right). \end{array}$$

$$X_{E1}=\left(\begin{array}{cccccc} \left\{y_{t-1}^{\left(1\right)}\right\} & \text{…} & \left\{y_{t-P}^{\left(1\right)}\right\} & f_{1}^{\left(1\right)} & \text{…} & f_{J}^{\left(1\right)}\\ \left\{y_{t-1}^{\left(2\right)}\right\} & \text{…} & \left\{y_{t-P}^{\left(2\right)}\right\} & f_{1}^{\left(2\right)} & \text{…} & f_{J}^{\left(2\right)}\\ \text{⋮} & \text{⋱} & \text{⋮} & \text{⋮} & \text{⋱} & \text{⋮}\\ \left\{y_{t-1}^{\left(E\right)}\right\} & \text{…} & \left\{y_{t-P}^{\left(E\right)}\right\} & f_{1}^{\left(E\right)} & \text{…} & f_{J}^{\left(E\right)} \end{array}\right)$$

$$X_{E2}=\left(\begin{array}{ccc} f_{1}^{\left(1\right)}\theta_{1}^{T} & \text{…} & f_{J}^{\left(1\right)}\theta_{1}^{T}\\ f_{1}^{\left(2\right)}\theta_{2}^{T} & \text{…} & f_{J}^{\left(2\right)}\theta_{2}^{T}\\ \text{⋮} & \text{⋱} & \text{⋮}\\ f_{1}^{\left(E\right)}\theta_{E}^{T} & \text{…} & f_{J}^{\left(E\right)}\theta_{E}^{T} \end{array}\right),\;y_{E}=\left(\begin{array}{c} \left\{y_{t}^{\left(1\right)}\right\}\\ \left\{y_{t}^{\left(2\right)}\right\}\\ \text{⋮}\\ \left\{y_{t}^{\left(E\right)}\right\} \end{array}\right)$$

$$\begin{array}{ll} B_{1}^{T} & =\left[\begin{array}{cccccc} A_{1} & \text{…} & A_{P} & c_{1} & \text{…} & c_{J} \end{array}\right]\\ B_{2}^{T} & =\left[\begin{array}{ccc} C_{1} & \text{…} & C_{J} \end{array}\right]. \end{array}$$

The dimensions of the above matrices are

$$\begin{array}{llll} X_{O1} & \in R^{\left(N_{O}-P\right)\times \left(SP+J\right)} & B_{1} & \in R^{\left(SP+J\right)\times S}\\ X_{O2} & \in R^{\left(N_{O}-P\right)\times JK} & B_{2} & \in R^{JK\times S}\\ X_{E1} & \in R^{\left(\sum_{e=1}^{E}\left(N_{e}-P\right)\right)\times \left(SP+J\right)} & y_{O} & \in R^{\left(N_{O}-P\right)\times S}\\ X_{E2} & \in R^{\left(\sum_{e=1}^{E}\left(N_{e}-P\right)\right)\times JK} & y_{E} & \in R^{\left(\sum_{e=1}^{E}\left(N_{e}-P\right)\right)\times S} \end{array}$$

The number of rows of y is denoted

$$N=\left(N_{o}-P\right)+\sum_{e=1}^{E}\left(N_{e}-P\right),$$

and therefore

$$\begin{array}{ll} y & \in R^{N\times S}\\ X & \in R^{N\times \left(SP+J+JK\right)}\\ B & \in R^{\left(SP+J+JK\right)\times S}. \end{array}$$

For large N, we follow common practice and ignore $L_{P}$, which corresponds to using the conditional likelihood (Box et al., 2008) (Section 7.1.2).

It is important to recognize that Equation A3 differs from traditional linear regression in that X includes the unknown parameter $\theta_{o}$. For future reference, we note that

$$X_{O2}B_{2}=\sum_{j=1}^{J}f_{j}^{\left(o\right)}\theta_{o}^{T}C_{j}^{T},$$

Maximum likelihood estimates are obtained by setting the first differential of the likelihood function to zero and solving the resulting equations. Usually, it is more convenient to perform this procedure on twice the logarithm of the likelihood function, which is

(A4)
$$2\;\log L=2\;\log L_{P}-SN\;\log\left(2\pi \right)-N\;\log|\Gamma |-tr\left[\Gamma^{-1}Z\right].$$

As discussed earlier, we ignore the differential of $L_{P}$.

To compute the differential, we will use the following standard identities (Magnus & Neudecker, 2001) (e.g., Sections 8.3 and 8.4).

(A5)
$$d\;\log|\Gamma |=tr\left[\Gamma^{-1}d\Gamma \right]$$

(A6)
$$d\Gamma^{-1}=-\Gamma^{-1}d\Gamma \Gamma^{-1}.$$

In addition, we use

$$tr\left[\Gamma^{-1}dZ\right]=-2tr\left[\Gamma^{-1}{\left(y-XB\right)}^{T}\left(dXB+XdB\right)\right].$$

Together, these identities give

(A7)
$$2dlogL=tr\left[\Gamma^{-1}\left(Z-N\Gamma \right)\Gamma^{-1}d\Gamma +2\Gamma^{-1}{\left(y-XB\right)}^{T}\left(dXB+XdB\right)\right]$$

The differential $d\theta_{o}$ is contained implicitly in dX. To make this relation more explicit, note that

(A8)
$$dXB=\left(\begin{array}{cc} 0 & dX_{O2}\\ 0 & 0 \end{array}\right)\left(\begin{array}{c} B_{1}\\ B_{2} \end{array}\right)=\left(\begin{array}{c} dX_{O2}B_{2}\\ 0 \end{array}\right)=\left(\begin{array}{c} \sum_{j}f_{j}^{o}d\theta_{o}^{T}C_{j}^{T}\\ 0 \end{array}\right),$$

and therefore

$$\begin{array}{ll} tr\left[2\Gamma^{-1}{\left(y-XB\right)}^{T}dXB\right] & =tr\left[2\Gamma^{-1}{\left(y_{1}-X_{O1}B_{1}-X_{O2}B_{2}\right)}^{T}\sum_{j}\left(f_{j}^{o}d\theta_{o}^{T}C_{j}^{T}\right)\right]\\ & =tr\left[2\left(v-U\theta_{o}\right)d\theta_{o}^{T}\right], \end{array}$$

where

$$\begin{array}{ll} v & =\sum_{j}C_{j}^{T}\Gamma^{-1}{\left(y_{1}-X_{O1}B_{1}\right)}^{T}f_{j}^{o}\\ U & =\sum_{j}\sum_{j^{\prime }}\left(f_{j^{\prime }}^{\left(o\right)T}f_{j}^{o}\right)C_{j}^{T}\Gamma^{-1}C_{j^{\prime }}. \end{array}$$

Substituting this into the first differential Equation A7 yields

(A9)
$$2dlogL=tr\left[\Gamma^{-1}\left(Z-N\Gamma \right)\Gamma^{-1}d\Gamma \right]+\left[2\Gamma^{-1}{\left(y-XB\right)}^{T}XdB\right]+tr\left[2\left(v-U\theta_{o}\right)d\theta_{o}^{T}\right]$$

In order for dlogL to vanish for all $d\Gamma ,dB,d\theta_{o}$, we must have

$$\begin{array}{ll} Z-N\Gamma & =0\\ {\left(y-XB\right)}^{T}X & =0\\ v-U\theta_{o} & =0, \end{array}$$

which in turn imply

(A10)
$$\hat{\Gamma }=Z/N$$

(A11)
$$\hat{B}={\left(X^{T}X\right)}^{-1}X^{T}y$$

(A12)
$${\hat{\theta }}_{o}=U^{-1}v,$$

where the caret symbol ˆ indicates a maximum likelihood estimate.

Equations (A10), (A11), (A12) are coupled because X depends on $\theta_{o}$, and therefore iterative methods must be used to obtain the solution. This iterative solution is facilitated by the fact that for given $\theta_{o}$, Equations A10 and A11 are the conditional maximum likelihood estimates of Γ and B, respectively. Therefore, substituting these equations into the likelihood function yields a function that depends only on $\theta_{o}$, resulting in a K‐dimensional minimization problem, a drastic reduction in the dimension of the optimization problem.

## Appendix B The Information Matrix

The covariance matrix of maximum likelihood estimates is obtained from the information matrix

(B1)
$${\left[I\right]}_{ij}=-E\left[\frac{\partial^{2}}{\partial \Theta_{i}\;\partial \Theta_{j}}\log L\right],$$

where we define the vector

(B2)
$$\Theta =\left(\begin{array}{c} \theta_{o}\\ vec\left(B\right)\\ vec\left(\Gamma \right) \end{array}\right).$$

In particular,

$$cov\left[\Theta \right]=I^{-1}.$$

The second derivatives appearing in Equation B1 may be computed by taking the second differential of logL.

Starting from Equation A7, we have

$$\begin{array}{ll} 2d^{2}\;\log L & =tr\left[d\Gamma^{-1}\left(Z-N\Gamma \right)\Gamma^{-1}d\Gamma \right]\\ & \;+tr\left[\Gamma^{-1}\left(dZ-Nd\Gamma \right)\Gamma^{-1}d\Gamma \right]\\ & \;+tr\left[\Gamma^{-1}\left(Z-N\Gamma \right)d\Gamma^{-1}d\Gamma \right]\\ & \;+tr\left[2d\Gamma^{-1}{\left(y-XB\right)}^{T}\left(dXB+XdB\right)\right]\\ & \;+tr\left[2\Gamma^{-1}{\left(-dXB-XdB\right)}^{T}\left(dXB+XdB\right)\right]\\ & \;+tr\left[2\Gamma^{-1}{\left(y-XB\right)}^{T}\left(dXdB+dXdB\right)\right]. \end{array}$$

To compute the associated expectations, we use the following facts:

$$\begin{array}{ll} E\left[Z\right] & =N\Gamma \\ dE\left[Z\right] & =E\left[{\left(y-XB\right)}^{T}\left(-dXB-XdB\right)\right]=0\\ E\left[y-XB\right] & =0. \end{array}$$

It follows from these facts that

$$\begin{array}{ll} E\left[2d^{2}\;\log L\right] & =tr\left[\Gamma^{-1}\left(-Nd\Gamma \right)\Gamma^{-1}d\Gamma \right]\\ & \;+tr\left[2\Gamma^{-1}{\left(-dXB-XdB\right)}^{T}\left(dXB+XdB\right)\right]. \end{array}$$

It is helpful to organize the terms in the following equivalent way:

$$\begin{array}{ll} E\left[2d^{2}\;\log L\right] & =-Ntr\left[\Gamma^{-1}d\Gamma \Gamma^{-1}d\Gamma \right]\\ & \;-tr\left[2\Gamma^{-1}B^{T}dX^{T}dXB\right]\\ & \;-tr\left[4\Gamma^{-1}dB^{T}X^{T}dXB\right]\\ & \;-tr\left[2\Gamma^{-1}dB^{T}X^{T}XdB\right]. \end{array}$$

Using Equation A8,

$$B^{T}dX^{T}XdB=\sum_{j}\sum_{j^{\prime }}C_{j}d\theta_{o}d\theta_{o}^{T}C_{j^{\prime }}^{T}\left(f_{j^{\prime }}^{\left(o\right)T}f_{j}^{\left(o\right)}\right).$$

Furthermore

$$dB^{T}X^{T}dXB=dB^{T}\sum_{j}X_{O}^{T}f_{j}^{\left(o\right)}d\theta_{o}^{T}C_{j}^{T},$$

where we define

$$X_{O}=\left(\begin{array}{cc} X_{O1} & X_{O2} \end{array}\right)$$

Substituting these into the expected second differential gives

(B3)
$$E\left[2d^{2}\;\log L\right]=-Ntr\left[\Gamma^{-1}d\Gamma \Gamma^{-1}d\Gamma \right]$$

(B4)
$$\;-2\sum_{j}\sum_{j^{\prime }}tr\left[\Gamma^{-1}C_{j}d\theta_{o}d\theta_{o}^{T}C_{j^{\prime }}^{T}\left(f_{j^{\prime }}^{{\left(o\right)}^{T}}f_{j}^{\left(o\right)}\right)\right]$$

(B5)
$$\;-4\sum_{j}tr\left[\Gamma^{-1}dB^{T}X_{O}^{T}f_{j}^{\left(o\right)}d\theta_{o}^{T}C_{j}^{T}\right]$$

(B6)
$$\;-2tr\left[\Gamma^{-1}dB^{T}X^{T}XdB\right]$$

We want to write this expression in terms of quadratic forms. Using standard properties of the trace, Equation B4 can be written as

$$-2d\theta_{o}^{T}\left(\sum_{j}\sum_{j^{\prime }}C_{j^{\prime }}^{T}\Gamma^{-1}C_{j}\left(f_{j}^{\left(o\right)T}f_{j^{\prime }}^{\left(o\right)}\right)\right)d\theta_{o}$$

For the other terms, we use the following identities from chapter 2.4 of Magnus and Neudecker (2001)

$$\begin{array}{ll} tr\left[ABCD\right] & ={\left(vec\left(D^{T}\right)\right)}^{T}\left(C^{T}⊗A\right)vecB\\ & ={\left(vecD\right)}^{T}\left(A⊗C^{T}\right)vecB^{T} \end{array}$$

where vec denotes the vectorization operator, ⊗ denotes the Kronecker product, and A,B,C,D refer to arbitrary matrices that are conformable in a way such that the product ABCD is defined.

Using these identities, Equation B3 becomes

$$-N{\left(dvec\Gamma \right)}^{T}\left(\Gamma^{-1}⊗\Gamma^{-1}\right)\left(dvec\Gamma \right),$$

Equation B5 becomes

$$-4{\left(dvecB\right)}^{T}\left(\sum_{j}\Gamma^{-1}C_{j}⊗X_{O}^{T}f_{j}^{\left(o\right)}\right)d\theta_{o}.$$

and Equation B6 becomes

$$-2{\left(dvecB\right)}^{T}\left(\Gamma^{-1}⊗X^{T}X\right)\left(dvecB\right).$$

Dividing these expressions by (−2) and collecting them into the appropriate matrix for the vector Equation B2 gives

(B7)
$$I=\left(\begin{array}{ccc} A & B & 0\\ B^{T} & C & 0\\ 0 & 0 & D \end{array}\right)$$

where

$$\begin{array}{ll} A & =\sum_{j}\sum_{j^{\prime }}C_{j^{\prime }}^{T}\Gamma^{-1}C_{j}\left(f_{j}^{\left(o\right)T}f_{j^{\prime }}^{\left(o\right)}\right)\\ B & =\sum_{j}C_{j}^{T}\Gamma^{-1}⊗f_{j}^{\left(o\right)T}X_{O}\\ C & =\Gamma^{-1}⊗X^{T}X\\ D & =N\left(\Gamma^{-1}⊗\Gamma^{-1}\right)/2. \end{array}$$

The covariance matrix for $\theta_{o}$ is

$$cov\left[\theta_{o}\right]={\left(A-BC^{-1}B^{T}\right)}^{-1}$$

Since C is a Kronecker product, its inverse is simple:

$$C^{-1}=\Gamma ⊗{\left(X^{T}X\right)}^{-1}$$

Furthermore, using

(W⊗X)(Y⊗Z)=WY⊗XZ,

we have

$$\begin{array}{ll} BC^{-1}B^{T} & =\left(\sum_{j}C_{j}^{T}\Gamma^{-1}⊗f_{j}^{\left(o\right)T}X_{O}\right)\left(\Gamma ⊗{\left(X^{T}X\right)}^{-1}\right)\left(\sum_{j^{\prime }}\Gamma^{-1}C_{j^{\prime }}⊗X_{O}^{T}f_{j^{\prime }}^{\left(o\right)}\right)\\ & =\sum_{j}\sum_{j^{\prime }}\left(C_{j}^{T}\Gamma^{-1}C_{j^{\prime }}\right)⊗\left(f_{j}^{\left(o\right)T}X_{O}{\left(X^{T}X\right)}^{-1}X_{O}^{T}f_{j^{\prime }}^{\left(o\right)}\right) \end{array}$$

Therefore, the covariance matrix for $\theta_{o}$ is

(B8)
$$\begin{array}{ll} cov\left[\theta_{o}\right] & ={\left(\sum_{j}\sum_{j^{\prime }}\left(\left(f_{j}^{\left(o\right)T}f_{j^{\prime }}^{\left(o\right)}\right)-f_{j}^{{\left(o\right)}^{T}}X_{O}{\left(X^{T}X\right)}^{-1}X_{O}^{T}f_{j^{\prime }}^{\left(o\right)}\right)\left(C_{j}^{T}\Gamma^{-1}C_{j^{\prime }}\right)\right)}^{-1}\\ & ={\left(\sum_{j}\sum_{j^{\prime }}f_{j}^{\left(o\right)T}\left(I-X_{O}{\left(X^{T}X\right)}^{-1}X_{O}^{T}\right)f_{j^{\prime }}^{\left(o\right)}\left(C_{j}^{T}\Gamma^{-1}C_{j^{\prime }}\right)\right)}^{-1} \end{array}$$

## Appendix C Regularized Estimation

We anticipate the need to regularize the estimation problem in situations involving small sample sizes. There is no unique way to regularize the problem, but a standard approach is to assume a prior distribution on θ of the form

$$p\left(\theta \right)∼N\left(\mu_{\theta },\Sigma_{\theta \theta }\right).$$

Then the posterior distribution of θ is

$$\begin{array}{ll} p\left(\theta_{o}|y_{e}y_{o}\theta_{e}B\right) & =p\left(y_{e}|\theta_{e}B\right)p\left(y_{o}|B\theta_{o}\right)p\left(\theta_{o}\right)\\ & ={\left(2\pi \right)}^{-NS/2}|\Gamma {|}^{-N/2}e^{-tr\left[\Gamma^{-1}Z\right]/2-{\left(\theta_{o}-\mu_{\theta }\right)}^{T}\Sigma_{\theta \theta }^{-1}\left(\theta_{o}-\mu_{\theta }\right)/2} \end{array}$$

The logarithm of the posterior times Equation 2 gives the regularized log‐likelihood function,

(C1)
$$2dlogL_{R}=-SN\;\log\left(2\pi \right)-N\;\log|\Gamma |-tr\left[\Gamma^{-1}Z\right]-{\left(\theta_{o}-\mu_{\theta }\right)}^{T}\Sigma_{\theta \theta }^{-1}\left(\theta_{o}-\mu_{\theta }\right).$$

In terms of the unregularized likelihood Equation A2,

$$2dlogL_{R}=2dlogL-{\left(\theta_{o}-\mu_{\theta }\right)}^{T}\Sigma_{\theta \theta }^{-1}\left(\theta_{o}-\mu_{\theta }\right).$$

The only difference between the unregularized and regularized log‐likelihoods Equations A4 and C1 is that there is an additional term depending on $\theta_{o}$ in the regularized version. Thus, the first order differentials associated with dB and dΓ equal their unregularized versions, while the differential associated with $d\theta_{o}$ is

$$tr\left[2\left(v-U\theta_{o}\right)d\theta_{o}^{T}-2\Sigma_{\theta \theta }^{-1}\left(\theta_{o}-\mu_{\theta }\right)d\theta_{o}^{T}\right].$$

This vanishes for all values of $d\theta_{o}$ if

$$\left(v+\Sigma_{\theta \theta }^{-1}\mu_{\theta }\right)-\left(U+\Sigma_{\theta \theta }^{-1}\right)\theta_{o}=0.$$

Solving for $\theta_{o}$ yields

$$\theta_{o}={\left(U+\Sigma_{\theta \theta }^{-1}\right)}^{-1}\left(v+\Sigma_{\theta \theta }^{-1}\mu_{\theta }\right).$$

As expected, the uninformative prior $\Sigma_{\theta \theta }^{-1}\to 0$ yields the unregularized solution. Also, in the limit a small uncertainty in the prior, $\Sigma_{\theta \theta }^{-1}\to \infty$ and $\theta_{o}$ reduces to the prior $\mu_{\theta }$.

For the second differential, regularization simply adds the term

$$d\theta_{o}^{T}\Sigma_{\theta \theta }^{-1}d\theta_{o}$$

Therefore, the information matrix relative to Equation B7 takes the form

$$I_{R}=\left(\begin{array}{ccc} A+\Sigma_{\theta \theta }^{-1} & B & 0\\ B^{T} & C & 0\\ 0 & 0 & D \end{array}\right)$$

Therefore, the covariance matrix of the regularized MLE of $\theta_{o}$ is

$$cov{\left[\theta_{o}\right]}_{R}={\left(\sum_{j}\sum_{j^{\prime }}f_{j}^{\left(o\right)T}\left(I-X_{O}{\left(X^{T}X\right)}^{-1}X_{O}^{T}\right)f_{j^{\prime }}^{\left(o\right)}\left(C_{j}^{T}\Gamma^{-1}C_{j^{\prime }}\right)+\Sigma_{\theta \theta }^{-1}\right)}^{-1}$$
